# In Vitro Genotoxicity Evaluation of PAHs in Mixtures Using Experimental Design

**DOI:** 10.3390/toxics11050470

**Published:** 2023-05-19

**Authors:** Rebecca Castel, Virginie Tassistro, Magalie Claeys-Bruno, Laure Malleret, Thierry Orsière

**Affiliations:** 1 Institut Méditerranéen de Biodiversité et Ecologie, Aix Marseille University, Avignon University, CNRS, IRD, IMBE, FR ECCOREV, ITEM, 13005 Marseille, France; rebecca.castel@imbe.fr (R.C.); virginie.tassistro@imbe.fr (V.T.); m.claeys-bruno@univ-amu.fr (M.C.-B.); 2 Laboratoire Chimie Environnement, Aix Marseille University, CNRS, LCE, FR ECCOREV, ITEM, 13545 Aix-en-Provence, France; laure.malleret@univ-amu.fr

**Keywords:** genotoxic equivalent factors, design of experiment, interaction effects, mixture hazard, comet assay, micronucleus

## Abstract

Settled dusts are sinks for environmental pollutants, including Polycyclic Aromatic Hydrocarbons (PAHs) that are ubiquitous, persistent, and carcinogenic. To assess their toxicity in mixtures, Toxic Equivalent Factors (TEFs) are routinely used and based on the hypothesis of additive effects, although PAH interactions may occur and remain an open issue. This study investigated genotoxic binary interaction effects for six PAHs in mixtures using two in vitro assays and estimated Genotoxic Equivalent Factors (GEFs) to roughly predict the genotoxicity of PAH in mixtures. The Design of the Experiment approach was used with the micronucleus assay for cytostasis and micronuclei frequency and the alkaline comet assay for DNA damage. GEFs were determined for each PAH independently and in a mixture. For the cytostasis endpoint, no PAHs interaction was noted. BbF and BaP had a synergistic effect on DNA damage. All the PAH interacted between them regarding chromosomal damage. Although the calculated GEFs were similar to the TEFs, the latter may underestimate the genotoxic potential of a PAH mixture. GEFs calculated for PAH alone were lower than GEFs for PAHs in mixtures; thus, mixtures induce greater DNA/chromosomal damage than expected. This research helps to advance the challenging issue of contaminant mixtures’ effects on human health.

## 1. Introduction

Nowadays, humans are exposed to more and more complex mixtures of contaminants in their daily environment. Children are the most vulnerable to this pollution, and it may impact their health long-term. They are especially exposed by ingestion, be it of food, water, soil, or dust [1]. Polycyclic Aromatic Hydrocarbons (PAHs) are both carcinogenic and ubiquitous in the environment and in domestic materials [2]. They are produced through incomplete combustion and may be sorbed onto particles, from ultrafine ones (<100 nm) to coarse particles that settle. Although the general population can be exposed to ultrafine particles through the respiratory route, the two other exposure routes, ingestion and dermal absorption have to be investigated thoroughly for settled dust. In particular, ingestion is considered to be the main exposure route to settled dust for children under 6 years old because of their exploratory behavior (hand-to-mouth behavior) [3,4]. The carcinogenicity of PAHs mainly resides in their capacity to form DNA adducts, thus damaging DNA and possibly inducing mutations [2]. Only benzo(a)pyrene (BaP) is classified as carcinogenic to humans, according to the International Agency of Research on Cancer (IARC) [5]. Therefore, it has been the most studied PAH in vivo and in vitro: by measuring benzo(a)pyrene diol epoxyde (BPDE), by investigating protein regulation, by quantifying its DNA damaging properties using the γ H2aX assay or the comet assay or by quantifying its chromosomal damaging properties using the in vitro or in vivo micronucleus assay [6,7,8,9,10,11]. However, BaP is never the only PAH present in a real-life context but is one target out of PAH complex mixtures. Other PAHs, such as naphthalene (Nap), benzo(b)fluoranthene (BbF), and dibenzo(a,h)anthracene (dBahA), are considered by IARC as possibly (BbF, Nap) or probably (dBahA) carcinogenic [5]. Some PAHs, in particular, phenanthrene (Phe) and pyrene (Pyr), are not classified as carcinogenic but are major contributors to the PAH contamination of particulate matter and settled dust [12]. As they are systematically found in environmental matrices, it is important to study if they may alter the effects of carcinogenic PAHs. Thus, research on the toxicity generated by mixtures of PAHs has been on the rise for the last two decades [13,14,15,16].

After absorption, PAHs are metabolized by phase I enzymes that catalyze their conversion into water-soluble metabolites, then phase II enzymes add hydrophilic groups to ease the elimination of the metabolites [17]. PAHs bind to the Aryl hydrocarbon receptor (AhR), which in turn will induce the production of the phases I and II enzymes [18]. In the case of BaP, this metabolism is responsible for its metabolic activation into its genotoxic metabolite BPDE [19]. The AhR signaling pathway may be induced by a wide range of organic contaminants, and interactions are expected when cells are exposed to a mixture of organic compounds [20]. The study of pollutant mixtures is necessary to progress into the recent concept of the exposome, but it is tricky, as there are hundreds of compounds on the market and an infinity of combinations of these compounds [21]. Nonetheless, for PAH mixtures, the Toxic Equivalent Factors (TEFs) are used to calculate a Toxic Equivalent Quantity (TEQ), being equal to the sum of the concentration of each PAH weighted by its corresponding TEF. This allows the comparison of different PAHs mixtures. The TEFs were established by Nisbet and LaGoy based on BaP being selected as the reference compound, on the similarity of the PAH’s toxic effects (qualitatively), on the similarity of the TEFs for different toxic endpoints, and on the additivity of effects of PAHs in mixtures. Most of the studies carried out to determine the TEFs focused on carcinogenicity in vivo or related toxic effects in vitro, such as PAH-DNA adducts [22]. The TEFs, routinely used in environmental risk assessment [23,24,25], are based on the hypothesis that PAH effects are additive, yet some recent studies indicated nonadditive effects in PAH mixtures [14,26,27,28]. Moreover, the European Food Safety Agency concluded that the TEFs could not be applied to PAH mixtures in food because of a lack of data on the oral carcinogenicity of individual PAHs [29]. Therefore, Audebert et al. and Tomasetig et al. calculated Genotoxic Equivalent Factors (GEFs) for PAHs in food without relying on carcinogenicity studies [30,31]. The aim of providing GEFs is to predict a genotoxic potential of a mixture by calculating its genotoxic equivalent quantity (GEQ) as the sum of the genotoxicity caused by each component i and related to its GEF (GEF_i_) and its concentration [i] in the mixture ($GEQ=\sum_{i=1}^{n}{GEF}_{i}\times \left[i\right])$. However, the established GEFs were calculated for each PAH studied independently. Thus, they do not take into consideration the possible interactions between PAHs when they are in mixtures.

Studies focusing on the toxicity of pollutants in mixtures generally dealt with binary equimolar mixtures, binary equitoxic mixtures, or environmental organic extracts with complex PAH mixtures and other unknown organic compounds [13,14,15], despite the fact that PAHs may display various effects depending on their proportion in a mixture [28]. Few are based on the design of experiments (DOE) approach [32]. DOE is a group of statistical methods that provide specific information for the selection and order of experiments [33]. It is used to identify the quantitative influence of chosen parameters by investigating the results (responses) of the experiments. The DOE may be used to study all the different interactions between factors (a full factorial design). To investigate these interactions with mixtures containing 6 PAHs, this would have consisted in studying the genotoxicity of 128 mixtures. In our study, the DOE’s benefits consist in performing a limited number of experiments, where combinations are chosen to study the influence of parameters (factors), by considering the interaction effects, which quantify the change in influence of one factor when another factor varies from a level to another. Thus, by limiting our study to principal effects and binary interaction effects, the total number of mixtures to investigate was 44. Overall, it is a useful approach to model possible non-additive effects of contaminants present in mixtures.

With the use of the DOE approach, we aimed to investigate if the in vitro genotoxic effects of six selected PAHs were, in fact, additive when weighted by their TEFs and when in a mixture. The PAHs studied were selected according to their carcinogenicity (Nap, BbF, BaP, dBahA) or their major environmental presence (Phe, Pyr). Their toxicities were assessed by using two in vitro tests that are routinely used to screen the genotoxicity of pharmaceuticals, cosmetics, and environmental chemicals [34,35,36]. The in vitro micronucleus assay is a reliable test for chromosomal damage and is recognized by OECD [37], and the in vitro comet assay is a sensitive test for DNA damage [38]. The in vitro micronucleus assay quantifies the frequency of micronuclei formed during cytokinesis. These micronuclei may be caused by chromosomal breakage or incorrect chromosomal segregation [37]. The comet assay quantifies the number of DNA fragments in the tail; these fragments may be caused by various DNA lesions, including oxidative strand breaks or by nucleotide excision repair, a mechanism to repair DNA adducts known to be formed by BPDE [39]. Adenocarcinoma gastric human cells (AGS) have successfully been used as a model to study the cyto- and genotoxicity of environmental mixtures to which children may be exposed by ingestion. As a matter of fact, nanoparticles and bioaccessible fractions of settled dust have been previously studied on this cell type [40,41]. Therefore, they were considered a relevant model for this study.

In our study, we aimed to determine whether the assumption that PAH effects are additive is true. Therefore, the DOE was coupled to two in vitro assays (comet/micronucleus) to cover cytotoxicity (cytostasis) and genotoxicity (DNA damage and chromosomal damage). GEFs were estimated for the five PAHs with BaP as the reference compound when they were studied independently and when in a mixture. The binary interaction effects were compared between the three endpoints. The GEFs were compared between the different experimental conditions and with the TEFs and other published GEFs.

## 2. Materials and Methods

### 2.1. Chemicals and Mixture Preparation

Goat antihuman Alexa Fluor^®^488, 40-6-Diamidino-2-phenylindole (DAPI), phalloidin-tetramethylrhodamine B isothiocyanate (TRITC), fetal bovine serum (FBS), Dulbecco’s PBS 1X and ProLong ™ Gold antifade mountant were provided by Life Technologies (Saint Aubin, France). Paraformaldehyde 4% PBS 1X was obtained from EMS (Hatfield, PA, USA). Bovine serum albumin fraction V was from Eurobio (Courtaboeuf, France). The PAHs (purity ≥ 96%) and all the other reagents were obtained from Sigma Aldrich (Lyon, France). The S9 mix kit was provided by Xenometrix (Allschwill, Switzerland); it included Aroclor 1254-induced lyophilized rat liver S9, G6P, NADP, and salt buffers. The S9 mixture was then prepared with 2.5% G6P, 10% NADP, 6.5% buffer salts, 10% S9 liver fraction, 21% H_2_O, and 50% PBS. PAH mixtures were prepared in DMSO at concentration levels allowing them to reach the target concentration of exposure by adding 2% of the PAHs mixtures to the culture medium.

### 2.2. Cellular Culture

Adherent human adenocarcinoma gastric cells (AGS) were maintained in Dulbecco’s Modified Eagle Medium (DMEM) at 37 °C with 5% of CO_2_. The DMEM was supplemented with 4.5 g/L of glucose, 2 mM of L-glutamine, 10% of fetal bovine serum, and 1% of penicillin–streptomycin. Both the AGS cells and the supplemented DMEM were provided by Cell Line Service (Eppelheim, Germany).

### 2.3. Cytokinesis-Block Micronucleus Assay

Cell viability was assessed using the cytokinesis-block proliferation index (CBPI), an index assessed on a number (No.) of 500 cells during the cytokinesis-block micronucleus (CBMN) assay analysis. It is calculated as follows:

(1) $$CBPI=\frac{\left[No.Mononucleated\;ȼ+\left(2\times No.Binucleated\;ȼ\right)+\left(3\times No.Multinucleated\;ȼ\right)\right]}{Total\;No.ȼ}$$

The CBPI obtained in the exposed and in the control conditions can then be used to assess a percentage of cytostasis:

(2) $$\%cytostasis=100-100\times (\frac{CBPIexposed-1}{CBPIcontrol-1})$$

The CBMN assay was performed to evaluate chromosomal damage [2,3]. Cells were seeded at 25.10^3^ cells/chamber in LabTek^TM^ 4 chamber slides (Lab-TekTM II Nalgene Nunc International, Villebon sur Yvette, France) 24 h prior to PAH exposure. Cells were exposed for 4 h to the tested mixtures (volume of 2%) in the presence of a 10% S9 mix. Negative control was performed using 2% DMSO and 10% S9mix. The positive controls were 10 ng/mL of mitomycin C (MMC) and 7.5 µM of BaP + 10% S9 mix. After 4 h, the medium was removed and replaced by a fresh medium for 20 h. Then the medium was renewed, and cytochalasin B (3 µg/mL) was added to each well to inhibit cell division after mitosis. Following a 24 h incubation, the medium was changed. After 2 h of rest, cells were washed with PBS and fixed with 4% of paraformaldehyde. Cells were incubated with a solution of PBS, 0.5% Triton X-100, and 2% BSA for 15 min to permeabilize the cells. Cells were then rinsed with PBS and 0.5% Triton X-100 before being incubated with 0.06 µg/mL of phalloidin-TRITC for 30 min to stain the cytoplasm. The cells were once again rinsed before a 10 min incubation with DAPI (1:50,000) to stain the nuclei. Finally, the slides were mounted in Prolong ™ Gold antifade and stored at 4 °C until analysis. The fluorescence microscope Zeiss Axio Imager A.2 (Zeiss SAS, Marly le Roi, France) was equipped with a filter combination and a Nikon camera (Melville, New York, NY, USA); the software was NIS-Elements. Scoring criteria were in accordance with the protocol described by Fenech [42]. The statistical significance of the differences in the micronucleated cell frequencies when comparing exposed conditions to their vehicle control was determined using the χ_2_ test. As this statistical significance is the most powerful criterion for a positive response in the CBMN test [OECD 487], χ_2_ values were used as results for the DOE approach [37].

### 2.4. Cytokinesis-Block Micronucleus Assay

The alkaline comet assay was performed to evaluate primary DNA damage [38]. Cells were seeded at 25.10^3^ cells/well in a 12 well-plate 24 h prior to PAH exposure. Cells were then exposed for 4 h to the tested mixtures and the negative control in the presence of 10% S9mix. After 4 h, the cells were trypsinized and pelleted. SuperForst^®^ Microscope slides (Thermo Scientific, Braunschweig, Germany) were pre-coated with 1.6% of normal melting point agarose and 0.8% of normal melting point agarose (Sigma-Aldrich affiliates Merck, KGaA, Darmstadt, Germany). The cellular pellets were resuspended in 1% low melting point agarose and spotted onto the slides. The positive control was performed by exposing AGS cells spotted onto slides to 125 µM of H_2_O_2_ for 4 min at 4 °C. Cells were then lysed by immersing the slides for 90 min at 4 °C in a lysis solution (2.5 M NaCl, 100 mM Na_2_EDTA, 300 mM NaOH, 10 mM Tris with 10% of DMSO and 1% Triton X-100; pH 10). After lysis, the denaturation was performed by immersing the slides for 20 min at 4 °C in a denaturation solution (300 mM NaOH and 1 mM EDTA). Electrophoresis was performed at 0.9 V/cm and 300 mA for 20 min at 4 °C. The slides were rinsed with a neutralizing buffer (4 mM Tris; pH 7.5) and water before being dehydrated in methanol. To evaluate DNA damage, the slides were stained with propidium iodide. The fluorescence microscope Zeiss Axio Imager A.2 (Zeiss SAS, Marly le Roi, France) was equipped with camera CCD Andor Zyla (Andor Bioimaging, Belfast, UK). The software was Komet 7.0 (Andor^TM^ Technology, Belfast, UK). For each slide, 100 cells were analyzed. All results are presented as a % increase in TailDNA as recommended by OECD 489 [43].

The effects of PAHs in mixtures were assessed on the %cytostasis to evaluate cytotoxicity, the %increase in TailDNA to evaluate the DNA damage induction, and the χ_2_ value to evaluate the chromosomal damage induction.

### 2.5. Experimental Factors and Domain of Interest

A quantitative study of factors was carried out. According to the literature and as mentioned in the introduction, six PAHs, noted X_i_ (i = 1–6), were selected as factors to build the factorial design matrix of experiments (Table 1). Two levels of concentration, coded lower and upper limits, were attributed to each factor (X_i_ = −1 and X_i_ = +1). The low and high concentrations were fixed to 1 µM and 10 µM in the absence of cytotoxicity. In the presence of cytotoxicity, the high concentration induced no more than 20% and 50% cytostasis for the comet assay and micronucleus assay, respectively. We calculated the TEQ of each mixture, and we assumed that a mixture with a given TEQ induced a %cytostasis similar to the one induced by BaP alone at a concentration equivalent to the latter given TEQ. Prior to that, we had studied the cytostasis induced by each PAH alone on AGS cells in a range of 2.5 nM to 10 µM. The concentrations of each PAH in the mixtures were chosen according to the above-mentioned requirements of the genotoxicity assays. The chosen concentrations correspond to the domain of interest of our study (Table 1).

We postulated that the results of each experiment, %cytostasis, %increase in TailDNA and χ_2_ value, were a linear combination of the main effects β_i_ (i = 1 to 6) and interaction effects β_ij_ (i, j = 1 to 6, i ≠ j) of each dimensionless variable X_i_. This corresponded to a linear model, and interactions between three or more factors were not considered.

(3) $$\eta =\beta_{0}+\beta_{1}X_{1}+\beta_{2}X_{2}+\beta_{3}X_{3}+\beta_{4}X_{4}+\cdots +\beta_{12}X_{1}X_{2}+\beta_{13}X_{1}X_{3}+\beta_{14}X_{1}X_{4}+\beta_{23}X_{2}X_{3}+\cdots +\beta_{56}X_{5}X_{6}+\varepsilon$$

where ε is a random error term representing whatever inaccuracy such a model can have. This model is valid only for the tested levels (−1 and +1) and, therefore, cannot be used for any interpolation or extrapolation.

In order to estimate the coefficients β, we performed a Rechtschaffner design [44]. It is a subset of the experiments from the full factorial design with 22 experiments (mixtures) for each genotoxicity assay. Some experiments were replicated (n = 3) to calculate the experimental variance and reinforce the reliability of the final model validations. Two experimental designs were created, one for the comet assay (Table 2) and one for the micronucleus assay (Table 3). Analysis was performed with the software AZURAD 1.3.4.

### 2.6. Determination of Genotoxic Equivalent Factors

To calculate GEFs, a model of least square regression without intercept was applied to different data sets: one for the CBMN data (χ_2_) of each PAH alone (four concentrations), one for the CBMN data (χ_2_) of PAHs in mixtures (two concentrations) and one for the Comet data (%increase TailDNA) of PAHs in mixtures (two concentrations). Thus, we obtained the coefficients for each PAH and scaled them on the BaP coefficient. The software was R version 4.0.5.

## 3. Results and Discussion

### 3.1. Cytostasis

**Figure 1:**
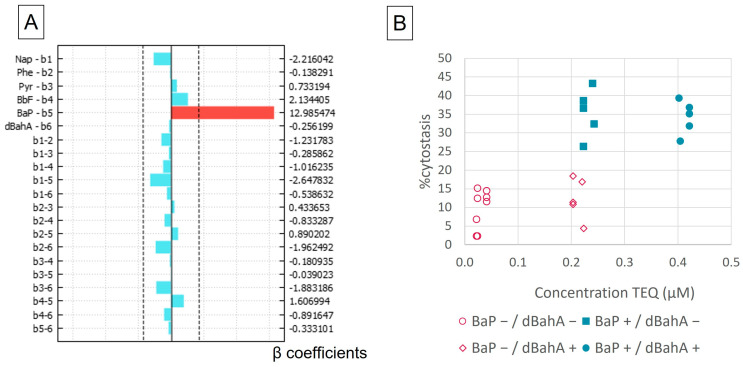
Cytostasis induction assessed with the CBPI on AGS cells, (**A**) graph representation of the effects, on right axis, value of the coefficients β, principal effects bi and binary interactions affects bij on the left, the dotted lines indicate the limits of significance, in blue the effects were not significant, in red the effects were significant; (**B**) %cytostasis of the 22 mixtures plotted against their Toxic Equivalent Quantity (TEQ) concentration, in blue the mixtures with high concentrations of BaP and in pink, mixtures with low concentrations of BaP.

Cytostasis was the first studied endpoint for 22 mixtures detailed in Table 3, as it is a necessary first step to ensure that the criteria to perform a CBMN analysis (%cytostasis < 50%) is met. Negative and solvent controls were not statistically different (less than 5%). Positive controls induced up to 50% of cytostasis. Figure 1A shows that BaP was the only factor with a significant effect on the response (%cytostasis). When BaP increased from 2.5 nM to 1 µM, an increase of 26% was noticed for the %cytostasis (26% = 2 × 12.98, 12.98 being the half-difference of the effect of BaP between the two levels). The TEQ is influenced by both BaP and dBahA, as they both have a TEF of 1. However, there was no significant effect of dBahA on %cysotasis (Figure 1A). In Figure 1B, the %cytostasis of each mixture was shown as a function of its TEQ. Two groups of mixtures can be observed: one group with less than 20% of cytostasis (data in pink) and one group with 30% to 40% of cytostasis (data in blue). The first group contained all the mixtures with low concentration levels of BaP (2.5 nM, see Figure 1B), whereas the second group contained all the mixtures with the upper concentration level of BaP (2 µM, see Figure 1B). The concentration levels of dBahA (0.2 or 2 µM) had no effect on the %cytostasis when BaP was set at its maximum level (2 µM). But there was a slight increase in the %cytostasis when dBahA was present at its upper level (2 µM) with BaP set at its lower level (2.5 nM) (Figure 1B). Overall, BaP was the only driver of cytostasis in our mixtures.

Staal studied equitoxic binary mixtures with different PAHs, including BaP, BbF, and dBahA [26]. When alone and up to 1 µM, BbF and dBahA influenced the cell cycle by increasing cells in S-phase. When in equitoxic mixtures with BaP, the percentages of cells in the S-phase were similar to the ones observed with BaP alone, suggesting that the cell cycle was mainly influenced by BaP. Gaskill and Bruce studied cell viability when cells were exposed to binary mixtures of different PAHs with BaP. They maintained constant the concentration of one PAH (1 mg/L) while increasing the concentration of the other PAH (0.25 mg/L to 10 mg/L) [28]. Binary mixtures of BaP and Phe gave similar results to the ones obtained with BaP alone. Binary mixtures of BaP and Pyr showed a slight increase in cell viability compared to BaP alone. Binary mixtures of BaP and BbF gave different results depending on which PAH was held at a constant concentration. When BaP was constant (1 mg/L), cell viability’s behavior depended on the concentration of BbF: it increased at lower concentrations (<2 mg/L) but decreased at higher concentrations (>5 ppm). When BbF was held constant, cell viability was similar to the results of BaP alone: it decreased when BaP’s concentration increased. Gaskill and Bruce’s work highlighted the need to study mixtures with different concentrations, as the effects may change depending on concentrations.

Although methodologies were different from ours (different cell lines with different metabolic capacities, different exposure duration, different endpoints), analogous conclusions can be drawn from these results and ours: even in mixtures with 6 PAHs, closer to environmental mixtures, BaP is the main PAH influencing the cell cycle and viability.

### 3.2. DNA Damage–Comet Assay

**Figure 2:**
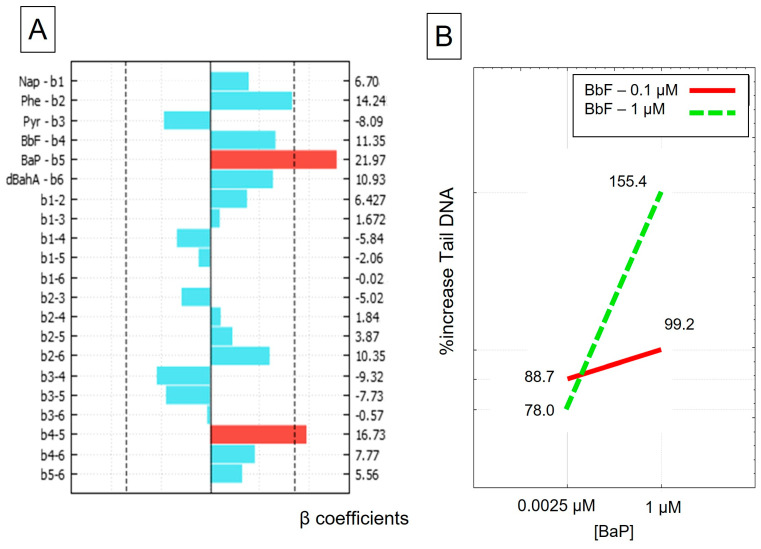
DNA damage assessed by the comet assay on AGS cells, (**A**) graphic representation of effects, on right axis, value of the coefficients β, principal effects bi and binary interactions affects bij on the left, the dotted lines indicate the limits of significance, in blue the effects were not significant, in red the effects were significant; (**B**) graphic representation of the binary interaction effect between BaP and BbF, concentrations on the X axis and %increaseTailDNA on the Y axis, BbF at 0.1 µM is represented by the solid red line and BbF at 1 µM is represented by the dotted green line.

DNA damage was one of the two genotoxic endpoints measured and was determined with the comet assay. Negative and solvent controls were not statistically different (only 3% of the difference in %TailDNA). The positive control induced a significant increase in DNA damage (up to 90% TailDNA). As shown in Figure 2A, we observed two significant effects: the principal effect of BaP alone (b_5_) and one binary interaction effect between BaP and BbF (b4–5). However, as BaP is also involved in a binary interaction effect, we cannot interpret the effect of BaP alone on DNA damage (Figure 2A). The binary interaction effect graphic in Figure 2B shows that when BaP was in the presence of 0.1 µM of BbF, there was a slight increase in response (from 88.7% to 99.2%, solid red line) (see Figure 2B). When BaP was in the presence of 1 µM of BbF, the BaP response soared from 77.9% to 155.4% (green dotted line). This type of response indicates there is a synergistic interaction between those two PAHs in the limit of the domain of interest.

A few recent studies also confirm the synergistic action of BbF when BaP and BbF were at an equimolar concentration of 1 µM. Thus, Staal et al. studied the formation of DNA adducts by a few PAHs at equitoxic binary concentrations. They observed that BbF and also dBahA, in a mixture with BaP, induced more DNA adducts than expected [26]. Tarantini et al. used the comet assay and quantified DNA adducts caused by equimolar binary mixtures of PAHs with BaP at 1 µM [14]. They observed that BbF and also dBahA, with BaP, slightly increased DNA damage, although they did not increase DNA damage when alone. On the contrary, binary mixtures of BaP and Pyr induced similar levels of DNA damage and DNA adducts than BaP alone, in accordance with our results showing no interaction effect between BaP and Pyr (b3–5). They then studied a range of concentration of BbF ([0.25–8 µM]) with 1 µM of BaP. They observed that there was an increase of DNA adducts when BaP was in presence of at least 1 µM of BbF, which is in good agreement with our results. Sevastonya et al. studied equimolar binary mixtures of BaP with BbF and BaP with dBahA in two different cell lines, HepG2 and HEL, and observed very different results depending on the cell type [13]. In HEL cells, the DNA adduct formation was inhibited for binary mixtures. However, HEL cells had low metabolic capacities on their own. In concordance with our results with AGS cells, the DNA adduct formation was amplified in HepG2 for binary mixtures of BaP (1 µM) with BbF or dBahA (1 µM). The inhibition effect of BbF or dBahA in the presence of BaP in HEL cells noticed by Sevastonya et al. pointed out that the genotoxicity of PAH mixtures and interaction effects could be different depending on the cell lines and their metabolic properties or the use of a metabolic activation system. Overall, our results were consistent with the findings of synergistic effect of BbF and no effect of Pyr in presence of BaP. Yet, in our study, no interaction effect between BaP and dBahA were evidenced. Furthermore, no interaction effect was encountered between BaP and Phe or Bap and Nap.

### 3.3. Chromosomal Damage–CBMN Assay

**Figure 3:**
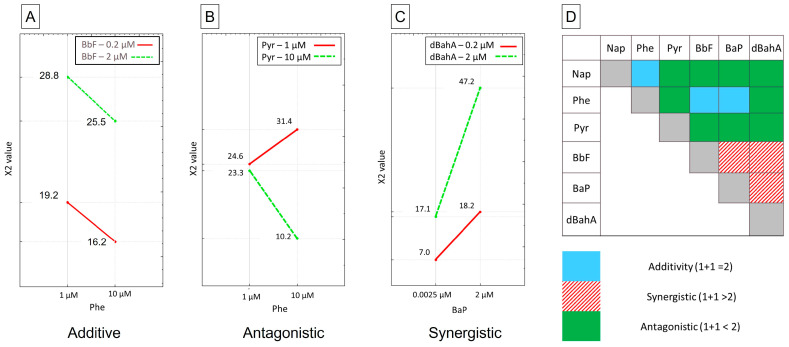
CBMN assay on AGS cells, (**A**): graphic representation of additive effects; (**B**): graphic representation of antagonistic effects; (**C**) graphic representation of synergistic effects; (**D**): binary interaction effects matrix, summarizing the interaction effect: in blue the additive effects, in hatched red the synergistic effects and in green the antagonistic effects.

Chromosomal damage was the second genotoxic endpoint measured and was assessed with the CBMN assay. Negative and solvent controls were not statistically different (χ_2_ < 3.84). Positive controls all induced a significant increase in micronuclei formation (χ_2_ > 12). Although only one binary interaction effect was significant with the comet assay, we observed 12 different binary interaction effects that were significant with the CBMN assay. We cannot assess the principal effects (b_i_) of each PAH independently, as they are all involved in binary interactions. All interactions are summarized in a binary interaction matrix (Figure 3D). Three binary interactions were the most statistically significant: between Phe and Pyr, between Phe and dBahA, and between BaP and dBahA; two being antagonistic (Phe and Pyr (Figure 3B, in green in Figure 3D); Phe and dBaha (in green in Figure 3D) and one synergistic (BaP and dBaha (Figure 3C, in hatched red in Figure 3D). Phe was the only PAH with additive effects (with Nap, BbF (see Figure 3A), and BaP (in blue in Figure 3D). If not, Phe had antagonistic interactions with Pyr and dBahA. Nap had mostly antagonistic interaction effects with the different PAHs, whereas all interaction effects with Pyr were antagonistic. Finally, each binary interaction between BbF, BaP, and dBahA was synergistic.

Peng et al. studied micronuclei formed by Nap, Phe, and Pyr alone in equimolar mixtures of these 3 (from 125 µM to 1000 µM) and the same equimolar mixtures with 20 µM of BaP on HepG2 cells [15]. They observed that only Nap had a significant increase of micronuclei alone at 125 µM, but not at higher concentrations. The equimolar mixture of Nap, Phe, and Pyr did not significantly increase the frequency of micronuclei or the cytostasis. Finally, the equimolar mixture with 20 µM of BaP had levels of cytostasis and micronuclei similar to 20 µM of BaP alone. It indicated that these PAHs had no effect on BaP cyto- and genotoxicity, while our own findings showed antagonistic interactions of BaP with Pyr or NaP when cells are exposed to mixtures of 6 HAPs. In another study investigating Nap, Phe, Pyr, and BaP, binary mixtures of BaP with Nap and BaP with Phe induced fewer micronuclei than BaP alone, suggesting, on the contrary, antagonistic interactions of BaP, with Phe or Nap [27]. The difference between these two studies resides in the concentration range; it was lower and close to ours in Muthusamy et al. ([5–15 µM] vs. much higher in Peng et al. [125–1000 µM] [12,24]. Gaskill and Bruce also studied binary mixtures on micronuclei formation [28]. They observed that BbF, Phe, and Pyr had antagonistic interactions with BaP. We only observed an antagonistic interaction between Pyr and BaP. Phe and BaP had additive effects, whereas BbF and BaP had a synergistic interaction. The results of the different studies (ours included) may differ due to the difference in experimental conditions, as the other studies were performed with binary/quaternary mixtures, and ours was performed with senary mixtures, thus being more representative of the real environment.

### 3.4. Determination of Genotoxic Equivalent Factors

The GEFs were determined for each PAH alone with the CBMN assay and for each PAH in mixtures with the comet assay and the CBMN assay (Table 4). When comparing the GEF calculated on CBMN data, we observed a lowering of the GEF of Nap and Pyr, from 0.1 and 0.01, respectively, to 0, indicating a decrease in genotoxicity induced by these two PAHs when in a mixture. Phe’s GEFs remained very low, 0.01 alone and 0.03 in the mixture. BbF’s GEF doubled, from 0.22 alone to 0.48 in the mixture. DbahA GEFs were close, 1.13 alone and 1.24 in the mixture. These results show the importance of determining GEFs for compounds when in the mixture, as interactions may alter the genotoxic potential of each compound. When comparing the GEFs calculated for PAHs in a mixture depending on the genotoxic assay, the ones calculated with the comet assay data were higher than the ones calculated with the CBMN assay data, except for dBahA. These results are coherent with the fact that DNA damage is more frequent than chromosomal damage, as a large proportion of them do not lead to DNA double-strand breaks. Nevertheless, the dBahA GEF doubled between the two assays, going from 0.61 in the comet assay to 1.24 in the CBMN assay. It could be due to the formation of a larger steric bulk than the other PAHs and could induce a higher frequency of double-strand breaks.

In agreement with the calculation of TEQ values, we can propose a linear equation to apportion the genotoxicity of PAH mixtures (GEQ value) by using the hereby determined mean GEFs for mixtures, as follows:

(4) $$GEQ=0.03\times \left[Nap\right]+0.05\times \left[Phe\right]+0.01\times \left[Pyr\right]+0.61\times \left[BbF\right]+0.93\times [dBahA]+1\times [BaP]$$

These GEFs could be used additionally to the TEFs in risk assessment to obtain the genotoxic potential of PAHs in mixtures.

When Nap was alone, it had a GEF of 0.2 but its GEF fell to 0–0.06 when in a mixture, close to the GEF of 0 determined by Tomasetig et al. and similar enough to the TEF of 0.001 set by Nisbet and LaGoy [19,34]. Phe and Pyr had GEFs of 0.01 when alone, but the GEF of Phe was slightly higher when in mixtures (up to 0.08), whereas the GEF of Pyr remained similar; both were one order of magnitude higher than their TEFs. The GEF of BbF was higher than the TEF in our study but in a lower range than the one determined by Tomasetig et al. using a γ H2AX endpoint. The TEF for BbF is offset at 0.1 and may underestimate BbF’s genotoxicity either alone (GEF of 0.22) or in the mixture (GEF of 0.74 for the Comet assay and 0.48 for the CBMN assay). As for dBahA, its GEF was 1.13 when alone and 0.61 to 1.24 when in the mixture. Once more, this GEF was in the same range as the TEF and γ H2AX GEF. When DOE analyses were performed on other-than-binary mixtures and with in vitro genotoxicity assays, GEFs were good predictors of the TEFS and better estimated the genotoxic potential of a mixture that TEF would underestimate. This approach could be applied to other pollutants with TEFs, such as dioxins, furans, and PCB [45,46]. To deepen the understandings and the prediction of PAH mixtures toxicities, forthcoming studies have to be performed with PAH mixtures, laboratory prepared or from the environmental origin, to compare the calculated GEQ and TEQ values with the results of genotoxicity assays.

## 4. Conclusions

To our knowledge, this is the first study focusing on binary interactions in mixtures containing more than two PAHs, thus being more representative of an environmental mixture, by using a DOE approach. Depending on the studied endpoint, interactions between PAHs varied. No interaction was observed for cytostasis. For DNA damage, only one interaction was observed: a synergistic one between BaP and BbF. In the case of chromosomal damage, there were many interactions between all six studied PAHs, and only a few interactions, specifically with Phe, were additive. Most were antagonistic, and only a few interactions, specifically with Phe, were additive. Of note, interactions between the higher molecular weight PAHs (BaP with BbF and dBahA) were synergistic. GEFS were determined for PAHs alone and PAHs in mixtures in order to better predict the genotoxic potential of a mixture. The increase in GEFs noticed when PAHs were in the mixture highlights the importance of studying PAHs in mixtures and interactions between them when considering biological effects. GEFs determined with both common (Hep3B) and uncommon (LS-174T, NCI-H358, and AGS) cell lines were of the same order of magnitude for BbF and dBahA, the most influential PAHs on genotoxicity in addition to BaP and slightly higher than the widely used TEFs. The TEFs may therefore underestimate the genotoxic potential of a given mixture to some extent. Studies are generally performed with homemade PAH mixtures, but the genotoxicity of real environmental mixtures was, to date, scarcely assessed [13]. The effect of the global matrix present in environmental samples must also be addressed in forthcoming studies.

Globally, our study emphasized that experimental conditions had to be chosen carefully when studying mixtures, as results differed depending on the cell lines, the metabolic properties of the cell lines, the use or the absence of exogenous metabolic activation, the level, and duration of the exposure, the chosen endpoint, the number of contaminants (binary, quaternary and senary mixtures) or even the data treatment. Moreover, this work calls for further combinations of the DOE approach with in vitro genotoxicity testing as an efficient statistical tool to study complex mixtures with numerous contaminants. Additional studies are needed to refine the method (cell lines, endpoints) and the data treatment/analysis to better predict health risks associated with exposure to environmental contaminants.

## Figures and Tables

**Table 1:** Experimental factors and concentration levels (µM) of each PAH for the genotoxicity assays Level −: Xi = −1; level +: Xi = +1.

Variable	Factors	Principal Effects	Comet Assay		Micronucleus Assay	
Xi		bi	Level −	Level +	Level −	Level +
X1	Naphthalene	b1	1	10	1	10
X2	Phenanthrene	b2	1	10	1	10
X3	Pyrene	b3	1	10	1	10
X4	Benzo(b)fluoranthene	b4	0.1	1	0.2	2
X5	Benzo(a)pyrene	b5	0.0025	1	0.0025	2
X6	Dibenz(a,h)anthracene	b6	0.1	1	0.2	2

**Table 2:** Experimental design, condition runs of the D-optimal design, and results for the comet assay. PAH concentrations levels for each mixture are given in µM. The TEQ for each mixture is also in µM.

Experiment	Nap	Phe	Pyr	BbF	BaP	dBahA	TEQ	%Increase in Tail DNA
1_1	1	1	1	0.1	0.0025	0.1	0.12	75.41
1_2	1	1	1	0.1	0.0025	0.1	0.12	55.16
1_3	1	1	1	0.1	0.0025	0.1	0.12	84.94
2	1	10	10	1	1	1	2.12	172.36
3	10	1	10	1	1	1	2.12	122.65
4	10	10	1	1	1	1	2.12	244.26
5	10	10	10	0.1	1	1	2.04	141.01
6	10	10	10	1	0.0025	1	1.13	109.41
7	10	10	10	1	1	0.1	1.23	118.05
8	10	10	1	0.1	0.0025	0.1	0.13	104.17
9	10	1	10	0.1	0.0025	0.1	0.13	117.40
10	10	1	1	1	0.0025	0.1	0.21	61.90
11	10	1	1	0.1	1	0.1	1.12	87.87
12	10	1	1	0.1	0.0025	1	1.02	60.52
13	1	10	10	0.1	0.0025	0.1	0.13	71.06
14	1	10	1	1	0.0025	0.1	0.21	73.10
15	1	10	1	0.1	1	0.1	1.12	92.09
16	1	10	1	0.1	0.0025	1	1.02	82.46
17	1	1	10	1	0.0025	0.1	0.21	60.65
18	1	1	10	0.1	1	0.1	1.12	77.90
19	1	1	10	0.1	0.0025	1	1.02	71.00
20	1	1	1	1	1	0.1	1.20	150.34
21	1	1	1	1	0.0025	1	1.11	78.98
22	1	1	1	0.1	1	1	2.01	80.99

**Table 3:** Experimental design, condition runs of the D-optimal design, and results for the CBMN assay. PAH concentrations levels for each mixture are given in µM. The TEQ for each mixture is also in µM.

Experiment	Nap	Phe	Pyr	BbF	BaP	dBahA	TEQ	%Cytostasis	χ_2_
1_1	1	1	1	0.2	0.0025	0.2	0.23	6.67	4.27
1_2	1	1	1	0.2	0.0025	0.2	0.23	−0.63	6.08
1_3	1	1	1	0.2	0.0025	0.2	0.23	2.20	4.76
2	1	10	10	2	2	2	4.22	45.11	39.69
3	10	1	10	2	2	2	4.22	34.92	50.78
4	10	10	1	2	2	2	4.22	31.68	56.01
5	10	10	10	0.2	2	2	4.05	27.60	5.01
6	10	10	10	2	0.0025	2	2.23	4.31	0.57
7	10	10	10	2	2	0.2	2.43	40.96	14.44
8	10	10	1	0.2	0.0025	0.2	0.24	9.96	8.58
9	10	1	10	0.2	0.0025	0.2	0.24	13.99	6.27
10	10	1	1	2	0.0025	0.2	0.41	12.59	1.26
11	10	1	1	0.2	2	0.2	2.23	26.18	0.62
12	10	1	1	0.2	0.0025	2	2.03	18.31	2.35
13	1	10	10	0.2	0.0025	0.2	0.24	14.99	8.49
14	1	10	1	2	0.0025	0.2	0.41	11.45	18.33
15	1	10	1	0.2	2	0.2	2.23	38.46	35.05
16	1	10	1	0.2	0.0025	2	2.03	10.74	29.60
17	1	1	10	2	0.0025	0.2	0.41	14.30	1.31
18	1	1	10	0.2	2	0.2	2.23	34.99	15.53
19	1	1	10	0.2	0.0025	2	2.03	11.30	30.72
20	1	1	1	2	2	0.2	2.4	43.09	26.86
21	1	1	1	2	0.0025	2	2.21	16.79	36.53
22	1	1	1	0.2	2	2	4.02	39.14	64.79

**Table 4:** Determination of the genotoxic equivalent factors (GEFs) of the six PAHs taken either alone or in mixture, and comparison with existing equivalent factors. GEFs for γ H2AX, determined on 3 cell lines (Hep3B, LS-174T, and NCI-H358), adapted from Tomasetig et al. [31].

	Single PAH			PAH in Mixtures	
PAH	TEF	GEF (γ H2AX)	GEF (CBMN Assay)	GEF (Comet-CBMN Assays)	Mean GEF for PAH Mixtures
	from [22]	from [31]	from Present Study		
Nap	0.001	0	0.10	0.06–0	0.03
Phe	0.001	0	0.01	0.08–0.03	0.05
Pyr	0.001	0	0.01	0.02–0	0.01
BbF	0.1	[0.69–1.46]	0.22	0.74–0.48	0.61
dBahA	1	[0.5–2.77]	1.13	0.61–1.24	0.93
BaP	1	1.00	1.00	1.00	1.00

## Data Availability

Data is contained within the article.
